# Patterns Among Same-Sex Spousal Couples: Diverse Sociocultural Representation in Alzheimer’s Research

**DOI:** 10.1093/geroni/igab046.1564

**Published:** 2021-12-17

**Authors:** Shana Stites

**Affiliations:** University of Pennsylvania, Philadelphia, Pennsylvania, United States

## Abstract

Emerging evidence shows that understanding characteristic patterns between study partners (SP) and subjects can inform initiatives to diversify representation of sociocultural groups in ADRD research. This study examined same-sex spousal dyads with the goal of identifying bellwethers of opportunities to build diversity in ADRD research. Descriptive analysis of The Aging, Demographics and Memory Study (ADAMS), which enrolled a subset of subjects from the Health and Retirement Study and a SP for each subject. Eight same-sex spousal couples were among 718 SP-subject dyads (1.1%). Gay men were 3 times as likely to be spousal SPs (n=6) than lesbians (n=2), even though women far outnumber men overall. Patterns in caregiving and other characteristics also differed. Same-sex couples are underrepresented in ADRD research. Patterns among those enrolled suggest masculine and feminine norms may drive research engagement. This is discussed in the context of increasing sociocultural diversity in ADRD research across key social groups.

